# Phylogenetic relationships and characterization of the complete chloroplast genome of *Rosa sterilis*

**DOI:** 10.1080/23802359.2021.1915200

**Published:** 2021-04-26

**Authors:** Huiqing Yan, Yanjing Liu, Zongmin Wu, Yin Yi, Xiaolong Huang

**Affiliations:** a School of Life Sciences, Guizhou Normal University, Guiyang, PR China; b Key Laboratory of State Forestry Administration on Biodiversity Conservation in Mountainous Karst Area of Southwestern China, Guizhou Normal University, Guiyang, PR China; c Key Laboratory of Plant Physiology and Development Regulation, Guizhou Normal University, Guiyang, PR China

**Keywords:** *Rosa sterilis*, Rosaceae, chloroplast genome, phylogenetic relationship

## Abstract

*Rosa sterilis* is an economically and important fruit that is extensively grown in Southwestern China. In this study, we determined the complete chloroplast genome of *R. sterilis* using high-throughput Illumina sequencing. The chloroplast genome of *R. sterilis* is 156,561 bp in size, containing a large single-copy region (LSC)(85,701 bp), a small single-copy region (SSC) (18,746 bp), and a pair of inverted repeat (IR) regions (each one of 26,057 bp). The overall GC content of the chloroplast genome is 37.23%, while the corresponding values of GC contents of the LSC, SSC, and IR regions are 35.20%, 31.37%, and 42.70%, respectively. The chloroplast genome of *R. sterilis* contains 130 genes, including 84 protein-coding genes, 37 tRNA genes, and 8 rRNA genes. The phylogenetic maximum-likelihood tree revealed that *Rosa chinensis* or *Rosa chinensis var. spontanea* is the closest related to *R. sterilis* in the phylogenetic relationship. This complete chloroplast genome can be further used for genomic studies, evolutionary analyses, and genetic engineering studies of the family Rosaceae.

*Rosa sterilis*, belonging to the Rosaceae family, is a perennial shrub and originates from the Karst areas of Guizhou Province (Liu et al. 2016). The fruits of *R. sterilis* have been widely consumed due to good flavor and bioactivities compounds, such as triterpenes, amino acids, flavonoids, and other phenylpropanoid derivatives (Luo et al. 2017). The aim of this study was to sequence, assemble, and characterized the complete chloroplast genome of *R. sterilis* to learn more about genetic knowledge*. R. sterilis* was planted under natural conditions in Guizhou Normal University, Guizhou province (Guiyang, China 26°42.408′ N; 106°67.353′ E). The leaves of *R. sterilis* were collected and deposited at the herbarium of Guizhou Normal University (GZNU-PRR-ML-01). The total genomic DNA of *R. sterilis* was extracted using the optimized CTAB method and sequenced using the high-throughput Illumina NovaSeq6000 Sequencing Platform System (Illumina Co., San Diego, CA).

Quality control was performed to remove low-quality reads and adapters using the FastQC (Andrews 2015). A total of 980,062 clean reads were obtained and the average value of Q30 is 90.88%, indicating the good quality for further analysis. The chloroplast genome was assembled using the SPAdes version 3.5.0 (http://cab.spbu.ru/software/spades/) and annotated using the CpGAVAS (Liu et al. 2012; Lapidus et al. 2014). The tRNA genes were further identified using ARWEN (http://mbio-serv2.mbioekol.lu.se/ARWEN/) and tRNAscan-SE (Lowe and Chan 2016; Laslett and Canbäck 2008). The physical map of the chloroplast genome of *R. sterilis* was generated using Organellar Genome DRAW. Clean reads were obtained with 150-bp paired-end reads. The complete chloroplast genome sequence together with corresponding annotations has been submitted to Genbank under the accession number of MW007387.

The chloroplast genome of *R. sterilis* is a circle molecular genome with the length of 156,561 bp, containing a large single-copy region (LSC) of 85,701 bp, a small single-copy region (SSC) of 18,746 bp, and a pair of inverted repeat (IR) regions of 26,057 bp in each one. The overall nucleotide consists of A (48,512 bp), T (49,745 bp), C (29,638 bp), and G (28,657 bp), with the total GC content is 37.23%, similar to other species in the *Rosaceae* (Huang and Cronk 2015). The largest GC content ratio was obtained in the IR regions (42.70%). The values of GC content ratio in the LSC and SSC region were 35.20% and 31.37% because the tRNA and rRNA genes extensively have fewer AT nucleotides (He et al. 2020). The chloroplast genome of *R. sterilis* comprised 130 genes, including 84 protein-coding genes, 37 transfer RNA (tRNA) genes, and 8 ribosomal RNA (rRNA) genes. Most genes for photosynthesis were localized in the LSC and SSC regions. In the IR regions, we determined seven protein-coding genes (*ycf2, ndhB, ycf1, rps12, rpl2, rpl23,* and *rps7*,), seven kinds of tRNA (*tRNA-CAT, tRNA-CAA, tRNA-GAC, tRNA-GAT, tRNA-TGC, tRNA-ACG,* and *tRNA-GTT*), and four kinds of rRNA (*rrn16 S, rrn23 S, rrn4.5S,* and *rrn5S*).

The chloroplast genomes of 18 species and varieties in the Rosaceae and *R. sterilis* were used to validate the phylogenetic position. The complete chloroplast genomes were aligned by MUSCLE version 3.8.31 (http://www.drive5.com/muscle/) method (Edgar 2004). The tree was constructed using the maximum likelihood with MEGA version 7 software, which the bootstrap value was calculated using 1000 replicates (Figure 1). The phylogenetic tree results showed that *R. sterilis* is clustered and closest related to *Rosa chinensis* (MH332770) or *Rosa chinensis var. spontanea* (*NC_038102*). The complete chloroplast of *R. sterilis* can be essential for genomic studies and genetic engineering studies of the family Rosaceae.

**Figure 1.**
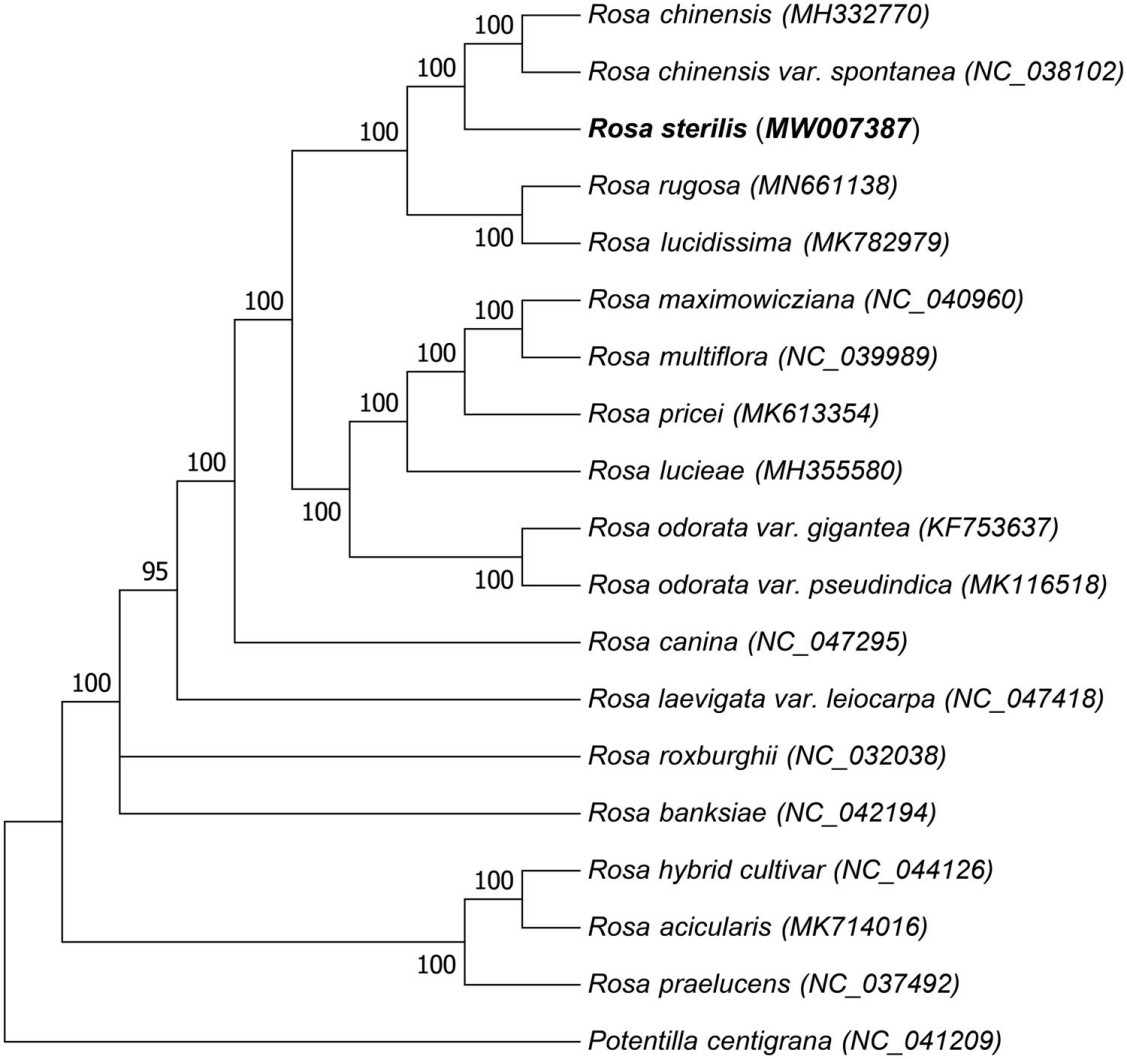
Maximum likelihood (ML) tree was constructed with other 18 genome sequences of Rosaceae. *Potentilla centigrana* was used as the out-group. Numbers at the right of nodes are bootstrap support values.

## Data Availability

The complete chloroplast genome sequences of *Rosa sterilis* together with corresponding annotations are available under the accession number MW007387 (http://www.ncbi.nlm.nih.gov/biosample/16561237). The chloroplast genome raw sequencing reads obtained by Illumina NovaSeq6000 sequencing are available at NCBI Sequence Read Archive (SRA) under the accession number SRR12967098. The Bioproject accession number is PRJNA672238 (http://www.ncbi.nlm.nih.gov/bioproject/672238) and the Biosample accession number is SAMN16561237 (http://www.ncbi.nlm.nih.gov/biosample/16561237).
